# Spatial and clinical epidemiology of spotted fever rickettsioses and ehrlichiosis, North Carolina, 2010–2019

**DOI:** 10.1371/journal.pntd.0013406

**Published:** 2025-08-13

**Authors:** Amanda Brown Marusiak, Dana A. Giandomenico, Brandon D. Hollingsworth, Neha V. Mokashi, Paul L. Delamater, Michael Reiskind, Alexis M. Barbarin, Carl Williams, Ross M. Boyce

**Affiliations:** 1 Department of Epidemiology, Gillings School of Global Public Health, University of North Carolina at Chapel Hill, Chapel Hill, North Carolina, United States of America; 2 Institute for Global Health and Infectious Diseases, University of North Carolina at Chapel Hill, Chapel Hill, North Carolina, United States of America; 3 School of Medicine, University of North Carolina at Chapel Hill, Chapel Hill, North Carolina, United States of America; 4 Department of Epidemiology and Biostatistics, Arnold School of Public Health, University of South Carolina, Columbia, South Carolina, United States of America; 5 Cornell Institute for Host Microbe Interaction and Disease, Cornell University, Ithaca, New York, United States of America; 6 Department of Biostatistics, Gillings School of Global Public Health, University of North Carolina at Chapel Hill, Chapel Hill, North Carolina, United States of America; 7 Carolina Population Center, University of North Carolina at Chapel Hill, Chapel Hill, North Carolina, United States of America; 8 Department of Geography, University of North Carolina at Chapel Hill, Chapel Hill, North Carolina, United States of America; 9 Department of Entomology and Plant Pathology, North Carolina State University, Raleigh, North Carolina, United States of America; 10 Division of Public Health, Communicable Disease Branch, Raleigh, North Carolina, United States of America; State Key Laboratory of Pathogen and Biosecurity, CHINA

## Abstract

**Background:**

North Carolina (NC) ranks among the top five states for spotted fever rickettsiosis (SFR) cases and second for ehrlichiosis in the U.S. Identifying geographic clusters of cases is important to elucidate disease risk and inform public health response, including resource allocation. This study examined geographic patterns of tick-borne disease incidence in NC over a 10-year period and modeled predictors of disease severity.

**Methodology/Findings:**

We analyzed 6,748 SFR and 1,216 ehrlichiosis cases reported to the NC Electronic Disease Surveillance System between January 2010 and December 2019. Average annual incidence was evaluated in two-year periods using global spatial autocorrelation (Moran’s I) and Local Indicator of Spatial Association. We found that ehrlichiosis clusters were detected in north and central NC as well as the coastal Tidewater region, with consistently high incidence in these areas. SFR clustering occurred in similar areas, with high and increasing incidence statewide. Severe cases of ehrlichiosis followed a similar pattern, while severe SFR clusters were distributed more broadly across the state. Additionally, Black/African-American individuals made up a greater proportion of both severe ehrlichiosis and SFR cases relative to non-severe cases. Regression models showed that known tick exposures were associated with lower odds of severe SFR. For SFR, treatment delays of 1–7 days were linked to severity, but delays >7 days were not. In contrast, delays >7 days for ehrlichiosis were associated with lower odds of severe disease.

**Conclusions/Significance:**

Associations found here between severity and treatment delay may reflect care-seeking behaviors, testing practices, and background seroprevalence. Geographic differences in disease incidence and severity warrant further investigation and future surveillance. Public health interventions should focus on the north-central and Tidewater regions, focusing on exposure risks awareness for outdoor activities and checking for ticks, which could impact treatment timing and ultimately reduce severity.

## Author summary

North Carolina has one of the highest rates of tick-borne diseases in the U.S, including spotted fever rickettsiosis (SFR) and ehrlichiosis. Understanding where cases of these diseases cluster across the state can improve disease prevention and resource allocation. This study analyzed thousands of SFR and ehrlichiosis cases reported in NC from 2010 to 2019, examining patterns of disease incidence and severity.

Ehrlichiosis clustered primarily in north-central NC and coastal Tidewater areas throughout the decade. SFR incidence was high and increased across the state over the study period, with even greater numbers of clusters in similar areas (north-central, Tidewater). Severe ehrlichiosis clusters overlapped with high-incidence areas, whereas severe SFR cases were more widely distributed.

Severe SFR was linked to known tick attachment and delayed treatment. Severe ehrlichiosis was not associated with exposure awareness, however, longer treatment delays for ehrlichiosis were associated with lower severity. This unexpected finding is possibly due to testing and treatment patterns unique to ehrlichiosis, which is less well-known than SFR.

These results highlight the need for targeted public health efforts in north-central NC and parts of the Tidewater region. Exposure awareness efforts should continue, emphasizing outdoor activities and the importance of checking for attached ticks.

## Background

Spotted fever rickettsiosis (SFR) and ehrlichiosis are tick-borne bacterial diseases that pose a threat to public health across much of the eastern and midwestern United States (US) [1,2]. Rocky Mountain spotted fever, caused by infection with *Rickettsia rickettsii*, is the prototypical – and most lethal – member of the spotted fever group [3]. In contrast, infection with species such as, *R. parkeri* is generally associated with less severe disease [4], while the pathogenicity of other commonly isolated bacteria such as *R. amblyommatis* is not well defined. Notably, there is cross reactivity on most serological tests, which makes it difficult to distinguish between species in human disease. Similarly, human monocytic ehrlichiosis is typically associated with infection with *Ehrlichia chaffeensis*, even though numerous additional species (e.g., *Ehrlichia ewingii, Ehrlichia muris*) have been identified as agents of human disease [5].

Although transmitted by different ticks, the initial symptoms of both diseases include fever, malaise, headache, and myalgia are both non-specific and relatively indistinguishable by history and physical examination alone. Progression to severe disease, most frequently associated with treatment delay [6,7], may result in the appearance of more prominent and specific symptoms (e.g., petechiae). Development of severe ehrlichiosis or SFR is associated with high rates of hospitalization and case fatality rates are reported between 1–10%, with fatal cases most likely to occur in children and older adults [1,3,5].

North Carolina (NC) reports the largest (n = 185, 15%) share of SFR cases nationally, a proportion that has steadily increased year-over-year [8]. Similarly, NC is the second leading state for ehrlichiosis incidence, accounting for 10% of cases (n = 135) in 2021 [9]. Factors that may contribute to the state’s high and increasing transmission of these diseases include a large rural population – second only to Texas [10] – with rapid expansion of metropolitan suburbs into previously wooded regions, relatively mild climate that facilitates and extended period of questing, and the high abundance of the tick vectors including *Amblyomma americanum* (Lone star tick) and *Dermacentor variabilis* (American dog tick) [11,12].

Several areas of the state have long been considered as high risk for tick-borne infection even as the data underlying these classifications have been derived from aggregate, county-level surveillance reports or focused research studies [13–18]. For a deeper understanding of disease risk across the state, more detailed analysis over space and time is necessary. By identifying specific areas of higher transmission or severe disease, surveillance and control efforts can be geographically targeted more efficiently. Additionally, examining data over a longer period can identify epidemiological trends and spatial patterns that may not be evident in short-term reports.

Therefore, the objective of this study was to identify geographic areas of SFR and ehrlichiosis risk and explore predictors of disease severity in NC over an extended timeframe. To achieve this goal, we analyzed case reports from the state public health reporting database spanning the period 2010–2019.

## Methods

### Ethics statement

The study was approved by the institutional review board of the University of North Carolina at Chapel Hill (IRB 22–0155). As a limited data set under CFR 45, Part 164.514 (e) written informed consent or waiver of HIPAA authorization was not required.

#### Data sources.

The NC Electronic Disease Surveillance System (NC EDSS) is a repository of state-mandated reportable communicable disease cases [19]. Reports are filed by healthcare providers, healthcare facilities and diagnostic laboratories. Confirmed and probable cases are investigated by a public health epidemiologist to collect additional information on individual demographic characteristics, potential exposures, and clinical outcomes. This investigation can include abstraction of medical records and/or direct interview with the patient. Eligible dates included a ten-year period from January 1, 2010, to December 31, 2019. Cases were grouped by calendar year based on the specimen collection date from the accompanying case report.

#### Case classification and exclusions.

NC EDSS classifies cases according to definitions from the Council of State and Territorial Epidemiologists case definitions for the respective time-periods (2008-present for SFR, 2000–2023 for ehrlichiosis) [20,21]. Both clinical information and laboratory results are considered in case classifications which include confirmed, probable and suspected. For almost all cases, laboratory results included immunofluorescent antibody testing for immunoglobulin G antibodies, the current standard diagnostic test for ehrlichiosis and SFGR. A small fraction of cases – 5.7% of Ehrlichia and 0.05% of SFGR– had polymerase chain reaction test results.

While cases of both human monocytic ehrlichiosis (i.e., ehrlichiosis) and human granulocytic ehrlichiosis (i.e., anaplasmosis) are reported in NC EDSS, only ehrlichiosis cases were included in this analysis. Based on laboratory test results, some individuals’ infection events were reported as both ehrlichiosis and anaplasmosis cases in separate records; in these instances (n = 24), we included only their ehrlichiosis record. A total of 250 anaplasmosis-only records were excluded.

#### Analysis.

Using the county of residence, each case was assigned to one of four topographical regions in the state, from west to east: (i) Blue Ridge Mountain, (ii) Piedmont, (iii) Inner Coastal Plain, and (iv) Tidewater. We defined severe cases as those with documentation of acute respiratory distress syndrome (ARDS), renal failure, disseminated intravascular coagulation (DIC), hospitalization, or death. Likely exposure setting categories were grouped from original case entries as: home, outdoors (woods or wilderness, farm, or body of water), other (work, camp, school, athletics, long-term care facility, incarceration, military, or international), and unknown (case record specified community or unknown). Tick exposure was categorized according to documentation of tick exposure or insect bite in each case record and/or notes, including: [1] tick attachment (documented “attachment”, “bite”, or “embedded” tick) [2], non-attached tick exposure or non-specific insect bite (no known attachment but possible exposure), or [3] no known tick exposure and [4] unknown tick exposure status.

We generated summary statistics of demographic, clinical, and exposure data for both ehrlichiosis and SFR cases. We conducted chi square and Wilcoxon rank sum tests to compare demographics between diseases. We also constructed logistic regression models for an outcome of severe disease across demographic and exposure variables. For these models, race was categorized as White, Black/African American, Asian, and other (Native Hawaiian or Pacific Islander, multiple races and other). Antibiotic treatment delay relative to specimen date was measured in three categories: none (on or before specimen date), 1–7 days and more than 7 days post specimen date. Immunocompromised status was determined by each case investigator during case review and informed by the CDC definition of immunosuppression, which includes a range of medical comorbidities and immunosuppressive treatments [22].

For the geospatial analysis, we generated ZIP code level maps of average annual incidence and case clusters using confirmed and probable cases of each disease. Data were grouped into five, two-year periods (e.g., 2010–2011, 2012–2013). We used population totals from 2020 census data as a denominator for incidence and removed one ZIP code with a very low population denominator (n = 10) that resulted in a very high outlier average annual incidence [23]. Category ranges for incidence on map legends align with surveillance reports for each disease from the North Carolina Department of Health and Human Services [4] We performed global spatial autocorrelation to evaluate the distribution (random, dispersed, or clustered) of ehrlichiosis and SFR case rates across the state during each period. Moran’s *I* was calculated for each period using the Queen case definition (those that share a common edge or vertex) for neighbors. Spatial clusters and outliers of zip codes were identified using the Local Indicator of Spatial Association (LISA), including high incidence surrounded by high incidence neighbors (high), low incidence surrounded by low incidence neighbors (low), high incidence with low incidence neighbors (high outlier), and low incidence with high incidence neighbors (low outlier).

Suspect cases were excluded from both regression and geospatial analyses due to the relatively low certainty of the underlying diagnoses. All analyses were performed using R statistical software (R Core Team 2021, version 4.2.1). We used R packages tigris, spdep and tmap packages for spatial analysis and map generation, respectively [24,25] We used a shapefile with North Carolina zip code polygons from the US Census Bureau [26].

## Results

**Fig 1:**
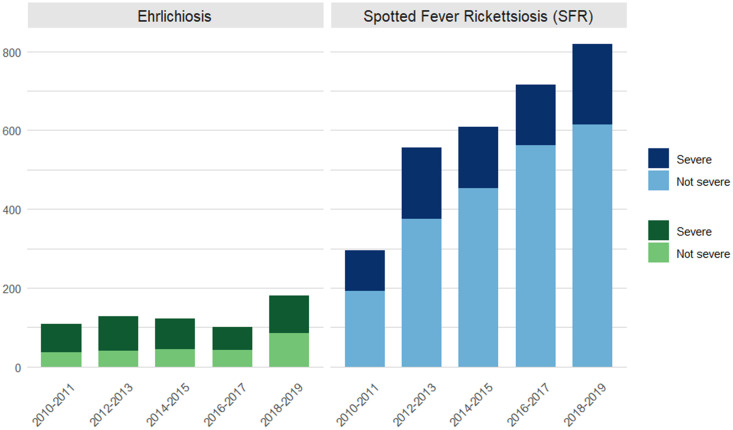
Cases of ehrlichiosis and Spotted Fever Rickettsiosis by 2-year study period and severity, NC EDSS 2010-2019.

Between January 2010 and December 2019, a total of 1,216 cases of ehrlichiosis and 6,748 cases of SFR were reported to NC EDSS (Fig 1 and Table 1). These included 151 (12.4%) confirmed, 747 (61.4%) probable and 318 (26.2%) suspect ehrlichiosis cases and 109 confirmed (1.6%), 4658 (69.0%) probable and 1981 (29.4%) suspect cases of SFR. While the median age for confirmed ehrlichiosis cases was higher than SFR (62 [49,71] vs 48 [33,64], p < 0.001), age was similar between probable (51 [33,65] vs. 48 [33,61]) and suspect (50 [36,63] vs. 52 [36,64]) cases (Table 1).

**Table 1:** Demographic characteristics of ehrlichiosis and SFR cases, NC EDSS 2010–2019.

	Ehrlichiosis (N = 1216)			SFR (N = 6748)		
Study Variables	Confirmed (N = 151)	Probable (N = 747)	Suspect (N = 318)	Confirmed (N = 109)	Probable (N = 4658)	Suspect (N = 1981)
**Age**						
Median [IQR]	62 [49, 71]	51 [33, 65]	50 [36, 63]	48 [33, 64]	48 [33, 61]	52 [36, 64]
Missing	0 (0%)	0 (0%)	1 (0.3%)	0 (0%)	1 (0.0%)	10 (0.5%)
**Sex**						
Female	61 (40.7%)	301 (40.7%)	143 (46.3%)	36 (33.3%)	1462 (31.6%)	756 (38.6%)
Male	89 (59.3%)	438 (59.3%)	166 (53.7%)	72 (66.7%)	3169 (68.4%)	1203 (61.4%)
Missing	1 (0.7%)	8 (1.1%)	9 (2.8%)	1 (0.9%)	27 (0.6%)	22 (1.1%)
**Race**						
Am. Indian/Al. Native	0 (0%)	1 (0.2%)	2 (1.1%)	1 (1.1%)	8 (0.3%)	9 (2.0%)
Asian	1 (1.0%)	9 (1.5%)	0 (0%)	0 (0%)	18 (0.6%)	6 (1.3%)
Black or African American	11 (10.7%)	102 (16.9%)	30 (16.0%)	5 (5.5%)	307 (10.0%)	77 (17.3%)
Multiple races	0 (0%)	1 (0.2%)	1 (0.5%)	0 (0%)	5 (0.2%)	1 (0.2%)
Native Hawaiian or Pac. Islander	0 (0%)	1 (0.2%)	0 (0%)	0 (0%)	5 (0.2%)	0 (0%)
White	91 (88.3%)	471 (78.1%)	153 (81.4%)	85 (93.4%)	2664 (87.0%)	345 (77.4%)
Other	0 (0%)	18 (3.0%)	2 (1.1%)	0 (0%)	54 (1.8%)	8 (1.8%)
Missing	48 (31.8%)	144 (19.3%)	130 (40.9%)	18 (16.5%)	1597 (34.3%)	1535 (77.5%)
**Hispanic ethnicity**						
Yes	1 (1.4%)	34 (7.6%)	5 (7.5%)	5 (6.9%)	121 (4.9%)	20 (8.1%)
No	69 (98.6%)	415 (92.4%)	62 (92.5%)	67 (93.1%)	2340 (95.1%)	228 (91.9%)
Missing	81 (53.6%)	298 (39.9%)	251 (78.9%)	37 (33.9%)	2197 (47.2%)	1733 (87.5%)
**Immunocompromised**						
Yes	23 (25.3%)	89 (19.3%)	1 (50.0%)	15 (20.3%)	309 (10.6%)	3 (8.1%)
No	68 (74.7%)	371 (80.7%)	1 (50.0%)	59 (79.7%)	2614 (89.4%)	34 (91.9%)
Missing	60 (39.7%)	287 (38.4%)	316 (99.4%)	35 (32.1%)	1735 (37.2%)	1944 (98.1%)
**Clinical outcome**						
Survived	142 (97.3%)	697 (98.7%)	38 (100%)	102 (99.0%)	4390 (99.6%)	296 (99.7%)
Died	4 (2.7%)	9 (1.3%)	0 (0%)	1 (1.0%)	16 (0.4%)	1 (0.3%)
Missing	5 (3.3%)	41 (5.5%)	280 (88.1%)	6 (5.5%)	252 (5.4%)	1684 (85.0%)
Severe disease*						
Yes	85 (56.7%)	302 (41.5%)	2 (4.9%)	40 (37.4%)	753 (16.6%)	12 (3.9%)
No	65 (43.3%)	426 (58.5%)	39 (95.1%)	67 (62.6%)	3790 (83.4%)	298 (96.1%)
Missing	1 (0.7%)	19 (2.5%)	277 (87.1%)	2 (1.8%)	115 (2.5%)	1671 (84.4%)
**Topographical Region**						
Blue Ridge Mountain	1 (0.7%)	44 (5.9%)	5 (1.6%)	14 (12.8%)	383 (8.2%)	53 (2.7%)
Piedmont	97 (64.2%)	521 (69.7%)	248 (78.0%)	65 (59.6%)	2652 (56.9%)	1111 (56.1%)
Inner Coastal Plain	24 (15.9%)	110 (14.7%)	41 (12.9%)	19 (17.4%)	908 (19.5%)	576 (29.1%)
Tidewater	29 (19.2%)	72 (9.6%)	24 (7.5%)	11 (10.1%)	715 (15.4%)	241 (12.2%)
**Travel history**						
Yes	15 (23.1%)	68 (18.8%)	0 (0%)	8 (13.3%)	417 (17.0%)	6 (28.6%)
No	50 (76.9%)	294 (81.2%)	1 (100%)	52 (86.7%)	2029 (83.0%)	15 (71.4%)
Missing	86 (57.0%)	385 (51.5%)	317 (99.7%)	49 (45.0%)	2212 (47.5%)	1960 (98.9%)
**Exposure setting**						
Home	29 (19.2%)	100 (13.5%)	0 (0%)	25 (22.9%)	687 (14.8%)	9 (0.5%)
Other	2 (1.3%)	33 (4.5%)	0 (0%)	4 (3.7%)	163 (3.5%)	1 (0.1%)
Outdoors	31 (20.5%)	153 (20.6%)	0 (0%)	39 (35.8%)	1104 (23.8%)	8 (0.4%)
Unknown	89 (58.9%)	455 (61.4%)	316 (100%)	41 (37.6%)	2689 (57.9%)	1947 (99.1%)
**Known tick exposure**						
Tick attachment	41 (27.2%)	125 (16.9%)	0 (0%)	33 (30.3%)	904 (19.5%)	5 (0.3%)
Non-attached tick exposure or non-specific insect bite	4 (2.6%)	17 (2.3%)	0 (0%)	9 (8.3%)	131 (2.8%)	2 (0.1%)
No known direct exposure	9 (6.0%)	87 (11.7%)	1 (0.3%)	9 (8.3%)	653 (14.1%)	6 (0.3%)
Unknown	97 (64.2%)	512 (69.1%)	315 (99.7%)	58 (53.2%)	2956 (63.7%)	1952 (99.3%)

* ARDS, DIC, renal failure, hospitalization, death.

A majority of cases with available self-reported racial data were White (80.1% ehrlichiosis, 84.5%) compared with Black or African American (15.9% of ehrlichiosis and 10.8% of SFR cases). Other racial categories (American Indian/Alaska Native, Native Hawaiian/Pacific Islander, multiple races) each comprised less than 2% of cases. A considerable proportion of all cases were missing race data (26.5% ehrlichiosis, 46.7% SFR). Of ehrlichiosis cases with available ethnicity data, 6.7% of all ehrlichiosis and 5.2% of all SFR cases were Hispanic.

Among confirmed and probable cases with available data, 20.3% of ehrlichiosis and 10.8% of SFR cases were categorized as immunocompromised. One ehrlichiosis case occurred in a pregnant person, which was classified as probable and was not severe, and eight SFR cases were reported in pregnant individuals, all probable cases, with two resulting in severe disease. There were 13 deaths (1.0%) associated with ehrlichiosis cases and 18 (0.3%) associated with SFR during the study period (Table 1). For each disease, less than 15% of suspect cases had adequate data available to assess severity.

Older age was associated with both severe ehrlichiosis (p < 0.001) and severe SFR (p < 0.001). (S1 Table). Immunocompromised status was also linked to severe outcomes for both diseases (ehrlichiosis p < 0.001, SFR p < 0.001). Gender was not associated with severity for either disease. Severity varied by race(ehrlichiosis p < 0.002, SFR p < 0.001) For ehrlichiosis, 78.7% of cases among Black individuals were severe compared to 55.5% among white individuals; for SFR, 48.5% of Black cases were severe versus 28.6% of white cases. Other race groups also had higher rates of severity relative to white cases, however sample sizes for these groups were very low.

### Logistic regression models of disease severity predictors

We found significantly lower odds of severe SFR among those with known home [aOR = 0.69, 95% CI: 0.50—0.94] or outdoor exposure [aOR = 0.55, 95% CI: 0.40—0.74] relative to those with an unknown exposure setting (Table 2). Having a known tick attachment was associated with lower odds of severe SFR [aOR = 0.61, 95% CI of 0.43—0.86]. No significant association was found between severe ehrlichiosis and exposure setting or any tick exposure status.

**Table 2:** Logistic regression results modeling odds of severe disease by exposure type and treatment status.

Independent variable	Adjusted OR [95% CI]	p-value
Ehrlichiosis		
Topographical region* *(ref: Blue Ridge Mountain)*		
Piedmont	2.04 [0.85, 5.31]	0.12
Inner Coastal Plain	**2.83 [1.05, 8.20]**	**0.05**
Tidewater	2.25 [0.80, 6.73]	0.07
Likely exposure setting* *(ref: unknown)*		
Home	0.58 [0.32, 1.04]	0.07
Outdoors	0.74 [0.29, 1.88]	0.5
Other	0.74 [0.44, 1.24]	0.8
Known tick exposure* *(ref: no known tick exposure)*		
Tick attachment	1.29 [0.63, 2.67]	0.5
Non-attached tick exposure or non-specific insect bite	0.40 [0.09, 1.55]	0.2
Unknown	1.07 [0.58, 1.97]	0.8
Any antibiotic treatment *(ref: no antibiotic treatment)*	2.09 [0.90, 5.17]	0.09
Doxycycline treatment *(ref: no doxycycline treatment)*	0.83 [0.43, 1.58]	0.6
Doxycycline treatment delay		
*From specimen date (ref: same day)*		
1–7 days post specimen date	0.92 [0.43, 1.94]	0.8
> 7 days post specimen date	**0.27 [0.08, 0.76]**	**0.02**
*From illness identification date† (ref: 0–3 days)*		
4–7 days post symptom onset	2.49 [1.00, 6.46]	0.06
8–14 days post symptom onset	2.39 [0.85, 7.05]	0.11
> 14 days post symptom onset	1.10 [0.40, 3.02]	0.9
Antibiotic treatment course *(ref: doxycycline only)*		
No doxycycline treatment	1.57 [0.80, 3.11]	0.2
Multiple antibiotics with delayed doxycycline	1.57 [0.38, 7.00]	0.5
Multiple antibiotics with immediate doxycycline	**18.6 [4.95, 122]**	**<0.001**
Spotted Fever *Rickettsia*		
Topographical region* *(ref: Blue Ridge Mountain)*		
Piedmont	0.95 [0.64, 1.43]	0.8
Inner Coastal Plain	1.04 [0.65, 1.67]	0.9
Tidewater	1.10 [0.68, 1.80]	0.7
Likely exposure setting* *(ref: unknown)*		
Home	**0.69 [0.50, 0.94]**	**0.02**
Outdoors	**0.55 [0.42, 0.73]**	**<0.001**
Other	0.63 [0.35, 1.09]	0.11
Known tick exposure* *(ref: no known tick exposure)*		
Tick attachment	**0.61 [0.43, 0.86]**	**<0.01**
Non-attached tick exposure or non-specific insect bite	0.55 [0.27, 1.06]	0.09
Unknown	0.71 [0.53, 0.96]	0.02
Any antibiotic treatment *(ref: no treatment)*	1.13 [0.68, 1.96]	0.5
Doxycycline treatment *(ref: no doxycycline)*	**0.62 [0.43, 0.89]**	**<0.01**
Doxycycline treatment delay		
*From specimen date (ref: same day)*		
1–7 days post specimen date	**1.54 [1.10, 2.14]**	**0.01**
> 7 days post specimen date	0.76 [0.37, 1.44]	0.3
*From illness identification date† (ref: 0–3 days)*		
4–7 days post symptom onset	1.07 [0.70, 1.61]	0.8
8–14 days post symptom onset	1.51 [0.93, 2.43]	0.09
> 14 days post symptom onset	1.09 [0.61, 1.89]	0.8
Antibiotic treatment course *(ref: doxycycline only)*		
No doxycycline treatment	**1.94 [1.33, 2.79]**	**<0.001**
Multiple antibiotics with delayed doxycycline	**5.06 [2.23, 11.7]**	**<0.001**
Multiple antibiotics with immediate doxycycline	**5.49 [2.98, 10.3]**	**<0.001**

Model covariates included age, race, immunosuppression and *doxycycline treatment (y/n).

†Models include only cases with known symptom onset date.

SFR cases treated with doxycycline had lower odds of severe disease [OR = 0.62, 95% CI of 0.43—0.89] compared to those not treated with doxycycline. No significant association was found between severe ehrlichiosis and doxycycline treatment. We measured the effect of treatment delay after testing among those who did receive antibiotic treatment and found lower odds of severe ehrlichiosis among cases with a treatment delay of more than 7 days following a test [OR = 0.27, 95% CI of 0.08—0.76] relative to no delay in treatment. No significant difference was detected for the 1-to-7-day treatment delay group relative to no delay. For SFR, a 1-to-7-day treatment delay was significantly associated with severe outcomes relative to no delay [OR = 1.54, 95% CI of 1.10—2.14]. No significant difference in severity was detected for SFR cases with more than 7 days delay in treatment relative to no delay following a test.

Race was evaluated as a potential effect measure modifier of predictors of disease severity listed in Table 2. Lack of variation in some predictor measures within racial categories prevented calculation of contrast measures and therefore full assessment of effect measure modification. However, where contrast measures were calculable, no effect measure modification by race was detected.

To address missingness in important model covariates including race, immunocompromised status and doxycycline treatment, we conducted a targeted sensitivity analysis of our regression results using multiple imputation. Notably, these variables exhibited a complex pattern of missingness that would require extensive modeling over various assumptions, which was beyond the scope of this study. However, we generated one such set of models focused on variables most likely to impact results. This sensitivity analysis S3 Table) showed results consistent in both magnitude and direction with results from the complete case approach in Table 2, supporting the robustness of our main findings.

### Geospatial analysis

**Fig 2:**
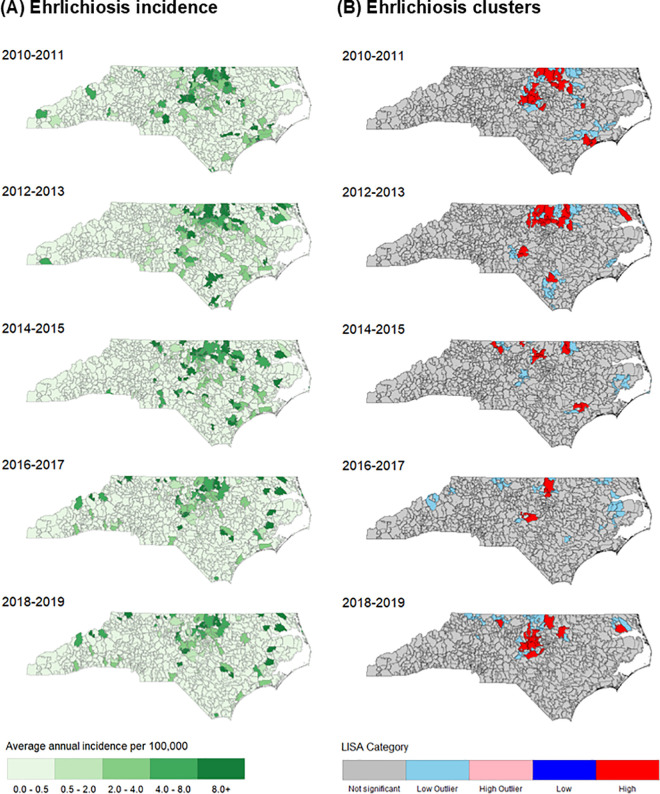
Average annual incidence (A) and local spatial autocorrelation (LISA) clusters (B) of confirmed and probable ehrlichiosis in North Carolina by ZIP code, 2010–2019. Maps generated using a TIGER/Line shapefile available from the US Census Bureau, https://www.census.gov/geographies/mapping-files/time-series/geo/tiger-line-file.html [26].

Moderate to high average annual incidence of ehrlichiosis (2 or more cases per 100,000 persons) was concentrated in the north-central part of the state throughout the full study period (Fig 2). Global Moran’s *I* analysis showed statistically significant ehrlichiosis clustering in all study periods except for 2016–2017, which had fewer clusters but retained similar regional geographic patterns to the other periods (S2 Table). LISA analysis consistently identified high cluster ZIP codes during every study period (n = 5–21), as well as many low-outlier clusters (n = 19–34) Most clusters were primarily detected in the north-central region, with fewer observed in the Tidewater region. The Mountain region in western NC generally reported few ehrlichiosis cases, with very few low outlier clusters detected only in 2016–2017 and 2018–2019.

**Fig 3:**
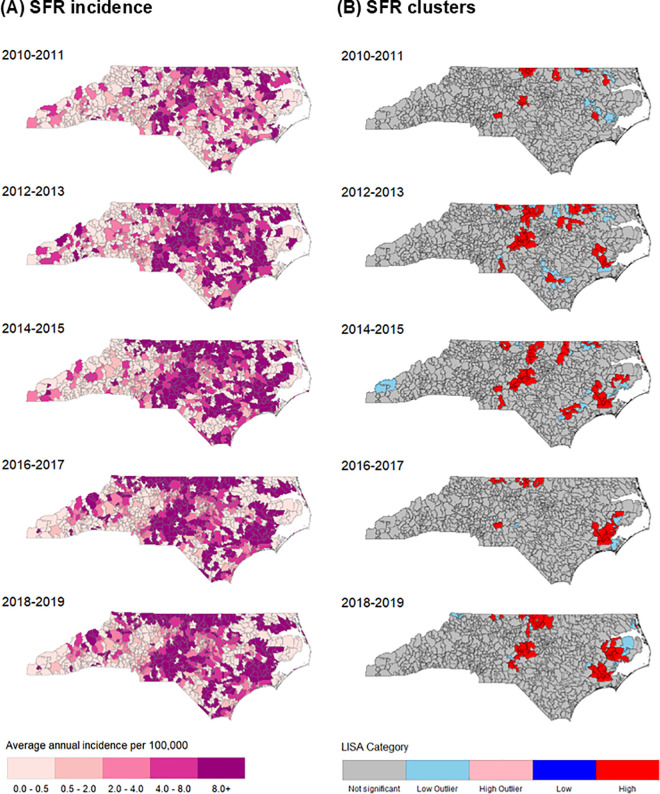
Average annual incidence (A) and local spatial autocorrelation (LISA) clusters (B) of confirmed and probable Spotted Fever Rickettsiosis (SFR) in North Carolina by ZIP code, 2010–2019. Maps generated using a TIGER/Line shapefile available from the US Census Bureau, https://www.census.gov/geographies/mapping-files/time-series/geo/tiger-line-file.html [26].

Over the full study period, SFR incidence was high and increased in many ZIP codes across the state (Fig 3). Similar to ehrlichiosis, cases were concentrated in the north-central part of the state as well as the Tidewater regions spanning the east and southern coast. Global Moran’s *I* analysis showed significant SFR clustering in all study periods (p < 0.001), with *I* ranging from 0.06 to 0.29. (S2 Table). Relative to ehrlichiosis, LISA analysis identified greater numbers of high SFR clusters in the Tidewater region for all but the first study period. No high clusters of SFR were identified in the Mountain region, and very few low outlier clusters occurred only in 2014–2015 and 2018–2019.

**Fig 4:**
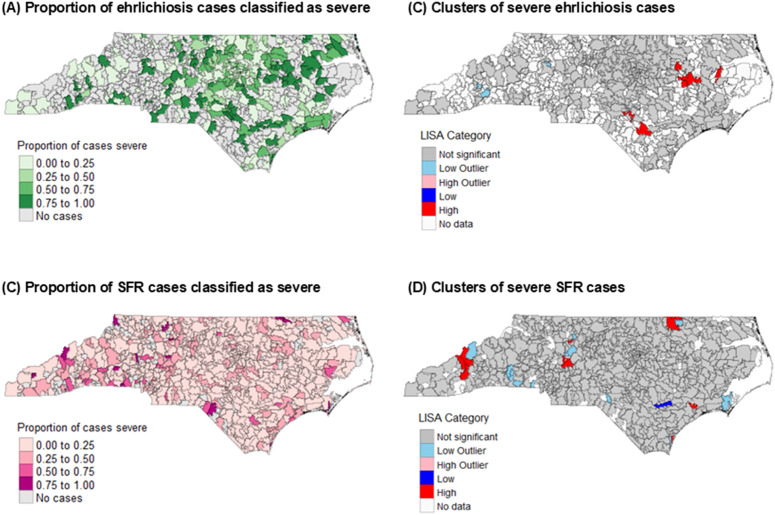
Severity of ehrlichiosis and Spotted Fever Rickettsiosis (SFR) in North Carolina by ZIP code, including proportion of cases classified as severe (A and C) and case clustering (B and D), 2010–2019. Maps generated using a TIGER/Line shapefile available from the US Census Bureau, https://www.census.gov/geographies/mapping-files/time-series/geo/tiger-line-file.html [26].

The proportion of ehrlichiosis that were classified as severe across the 10-year period was high in many ZIP codes across the state. High clusters of severe ehrlichiosis occurred in central areas of the state (n = 7), with low-outliers occurring in the Mountain region (Fig 4). Compared to ehrlichiosis, fewer ZIP codes had a high proportion of SFR cases that were severe. LISA analysis showed some clusters of severe SFR in various groupings across all regions of the state. Moran’s *I* for severe ehrlichiosis clustering was 0.12 (p < 0.01) and 0.06 (p < 0.01) for SFR (S2 Table).

Lastly, we evaluated clustering separately for high season (April through October) and low season (November through March) but did not find markedly different spatial patterns from the combined analysis. There were few low season clusters for ehrlichiosis, and SFR low season clustering tended to be only in southeastern portions of the state (S1 and S2 Figs).

## Discussion

Our ten-year analysis highlights important epidemiological and spatiotemporal trends in SFR and ehrlichiosis across NC, a high incidence state with a substantial burden of disease. We found focal, geographically stable incidence of ehrlichiosis in areas of north-central NC and the Tidewater region. Similar patterns were seen in SFR incidence, with greater numbers of high clusters in both regions. While clusters of severe ehrlichiosis were also found in central and Tidewater areas, severe SFR did not have a clear geographic pattern. Although older age was associated with severe disease, our findings regarding treatment delays diverge by disease and likely require a more nuanced interpretation. Overall, the results of our study raise novel, hypothesis-generating questions to guide future investigations towards public health interventions and efforts to reduce morbidity and mortality related to SFR and ehrlichiosis.

From a spatial standpoint, we found evidence of increased incidence of severe ehrlichiosis in the eastern regions. It is not clear what factors may contribute to the increased risk, but given lower median income of the region [27], unmeasured socioeconomic issues could limit access, such that severe cases that demand healthcare interventions make up a larger portion of cases. We also found clusters of severe disease for both ehrlichiosis and SFR in the north-central part of the state. This could be due to a compound effect of these areas being more rural with older populations, resulting in both increased likelihood of exposure and biological risk for severe disease. While it is well established that *R. rickettsii* is the most virulent of the SFR [3,28], less is known about *Ehrlichia* species. If, for example, *E. chaffeensis* is more likely to cause severe disease than *E. ewingii* as suggested by one study [29], local differences in distribution may contribute to the spatial findings here. Further investigation of pathogen dynamics would require species-specific testing, which is not yet widely accessible for diagnostic purposes.

To our knowledge, this is the first study to explore geographic differences in disease severity at the state level. Although our data set cannot establish causality, it clearly highlights the need to examine trends in vector, pathogen species, and case distribution to explain these regional differences.

At the individual level, we found that those with known possible exposure at home or outdoors had approximately half the odds of severe SFR compared to those with no reported knowledge of exposure setting. This may be because these individuals were more aware of the possibility of exposure, potentially seeking care more immediately and reporting the exposure to their provider, resulting in more timely treatment. This is also true for those with documented tick attachment, who had nearly half the odds of severe disease relative to individuals with no known exposure. We also observed increased risk of severe disease among self-reported Black or African American as compared to White cases. Like many other health conditions, there may be longstanding racial disparities with regard to care, as with regional variation in access. However, given the prominence of skin findings including early tick attachment or the later dermatological manifestations – the petechial rash associated with SFR or the more macular rash often seen with ehrlichiosis – poor clinical practice in recognizing these signs of disease in darker skin tones may contribute to delayed recognition.

In contrast to previous studies, our analysis of the associations between severe disease and treatment delays was not as clear. While we found that a treatment delay of 1–7 days after testing in SFR cases was associated with more severe illness, we also observed decreased risk with longer delays. This may be the result of misclassification of cases due to seropositivity resulting from prior exposure or infection with less pathogenic bacteria in which case treatment may not have been necessary. For example, previous research has estimated a lower risk for hospitalization in patients infected with *R. parkeri* [4], which is known to be circulating in the state [30]. For ehrlichiosis, similar trends were found for treatment delays from symptom onset, with lower odds of severe disease for treatment initiated after three days post-onset. This trend could reflect care-seeking behavior and testing patterns rather than clinical outcomes related strictly to treatment timing as found in current literature [6,28]. Cases that progressed to severe illness may have already been quite ill when care was initially sought, or when SFR or ehrlichiosis was suspected, at which point treatment was initiated before or concurrently with testing.

Our study has several strengths including its large sample size of all reported cases to the state over a decade. The geospatial analysis we conducted provides more granular assessment of SFR and ehrlichiosis risk compared to larger county-based and statewide surveillance reports. The study also has important limitations. First, cases were geolocated using their county of residence and home ZIP code as opposed to the county or ZIP code of their exposure, thus spatial assessment is not a perfect measure of exposure location, introducing possible geographic misclassification. While this study was comprised of all available formal case reports to the state, we could not capture the many individuals who were likely tested and not reported [31], as well as subclinical infections that are not tested, and symptomatic individuals that are never appropriately tested [18].

For the spatial analysis, given the exploratory nature of our study, we limited our analysis to two widely-used metrics (Moran’s I and LISA) and a single neighborhood definition; future work on the spatial patterns of SFR and ehrlichiosis may benefit from considering additional metrics. Our findings were also limited by the lack of paired entomological data that would allow us to assess risk in relation to vector density and distribution. Given the rapid changes in the geographic range of vectors, this will be an important element of surveillance efforts [32]. Finally, because we did not account for potential spatial autocorrelation in the logistic regression analysis, we were conservative in interpreting the strength of these results (given that the presence of spatial autocorrelation can lead to confidence intervals that are too narrow). Despite these limitations, our analyses provide important waypoints for future investigation and targeted public health interventions.

## Conclusion

This study reveals significant epidemiological and spatial trends in SFR and ehrlichiosis over a decade in North Carolina, a state with substantial burden of disease. We found both ehrlichiosis and SFR clusters in the north-central areas of the state as well as the Tidewater coastal region. Our analysis also identified clusters of severe disease across the state that varied by disease, which should be further explored with consideration to rurality of these areas as well as age, socioeconomic status and healthcare access. Additionally, our analysis highlights disparities in severe disease risks, particularly related to exposure awareness and race. Public health mitigation efforts should prioritize high-risk areas as identified here. Education of healthcare providers and the general public regarding exposure risks and prompt-care seeking should be essential strategies for severe disease prevention.

## Supporting information

S1 Table Disease severity across demographics, confirmed and probable cases of ehrlichiosis and Spotted Fever Rickettsioses (SFR), North Carolina, 2010–2019. (DOCX)

S2 Table Spatial cluster categories and global Moran’s *I* values for ehrlichiosis and Spotted Fever Rickettsiosis (SFR) in North Carolina by ZIP code, 2010–2019. (DOCX)

S3 Table Sensitivity analysis of logistic regression models for predictors of severe ehrlichiosis and SFR in North Carolina, 2010–2019. (DOCX)

S1 Fig Biannual high season (April through October) and low season (November through March) clusters of ehrlichiosis in North Carolina by ZIP code, 2010–2019. (DOCX)

S2 Fig Biannual high season (April through October) and low season (November through March) clusters of Spotted Fever Rickettsiosis in North Carolina by ZIP code, 2010–2019. (DOCX)
