# Supplementary material for: Spatial and clinical epidemiology of spotted fever rickettsioses and ehrlichiosis, North Carolina, 2010–2019
Source: PLoS Negl Trop Dis. 2025 Aug 13;19(8):e0013406. doi: 10.1371/journal.pntd.0013406 (PMC12364335; doi:10.1371/journal.pntd.0013406)
Supplement: S1 Table — (DOCX) [file pntd.0013406.s001.docx]

**Table 1. Disease severity across demographics, confirmed and probable cases of ehrlichiosis and Spotted Fever Rickettsioses (SFR), North Carolina, 2010-2019**

|  | **Ehrlichiosis** | | | **SFR** | | |
| --- | --- | --- | --- | --- | --- | --- |
| **Study Variables** | **Yes (N=389)** | **No (N=251)** | **Χ^2^  p-value** | **Yes (N=805)** | **No (N=2199)** | **Χ^2^  p-value** |
| **Age** |  |  |  |  |  |  |
| Mean (SD) | 54.6 (19.8) | 46.5 (19.3) | **<0.001** | 51.6 (20.4) | 45.5 (18.7) | **<0.001** |
| Median [IQR] | 59 [42, 69] | 49 [31, 61] |  | 54 [36, 68] | 46 [32, 60] |  |
| Missing | 0 (0%) | 0 (0%) |  | 1 (0.1%) | 0 (0%) |  |
| **Gender** |  |  |  |  |  |  |
| Female | 142 (57.0%) | 107 (43.0%) | 0.147 | 235 (25.1%) | 702 (74.9%) | 0.191 |
| Male | 243 (63.1%) | 142 (36.9%) |  | 562 (27.4%) | 1486 (72.6%) |  |
| Missing | 4 | 2 |  | 8 | 11 |  |
| **Race** |  |  |  |  |  |  |
| American Indian/Alaska Native | 2 (100%) | 0 (0%) | **0.002** | 2 (50.0%) | 2 (50.0%) | **<0.001** |
| Asian | 5 (83.3%) | 1 (16.6%) |  | 6 (40.0%) | 9 (60.0%) |  |
| Black or African American | 70 (78.7%) | 19 (21.3%) |  | 99 (48.5%) | 105 (51.5%) |  |
| Multiple races | 0 (0%) | 1 (100%) |  | 1 (25.0%) | 3 (75.0%) |  |
| Native Hawaiian or Pacific Islander | 1 (100%) | 0 (0%) |  | 2 (50.0%) | 2 (50.0%) |  |
| White | 231 (55.5%) | 185 (44.5%) |  | 520 (28.6%) | 1320 (71.4%) |  |
| Other | 8 (61.5%) | 5 (38.5%) |  | 14 (35.0%) | 26 (65.0%) |  |
| Missing | 72 | 40 |  | 161 | 732 |  |
| **Hispanic ethnicity** |  |  |  |  |  |  |
| Yes | 16 (59.3%) | 11 (40.7%) | 0.895 | 33 (25.1%) | 61 (64.8%) | 0.152 |
| No | 211 (56.0%) | 166 (44.0%) |  | 471 (27.7%) | 1228 (72.3%) |  |
| Missing | 162 | 74 |  | 301 | 910 |  |
| **Immunocompromised** |  |  |  |  |  |  |
| Yes | 81 (80.1%) | 20 (19.9%) | **<0.001** | 150 (58.4%) | 107 (41.6%) | **<0.001** |
| No | 173 (46.4%) | 200 (53.6%) |  | 408 (18.7%) | 1778 (81.3%) |  |
| Missing | 135 | 31 |  | 247 | 314 |  |
| **Topographical Region** |  |  |  |  |  |  |
| Mountain | 12 (34.3%) | 23 (65.7%) | **<0.001** | 72 (25.8%) | 207 (74.2%) | **0.004** |
| Piedmont | 242 (58.6%) | 171 (41.4%) |  | 452 (24.8%) | 1371 (75.2%) |  |
| Inner Coastal Plain | 89 (76.7%) | 27 (23.3%) |  | 161 (32.3%) | 338 (67.7%) |  |
| Tidewater | 46 | 30 |  | 120 | 283 |  |
| **Known tick exposure** |  |  |  |  |  |  |
| Tick attachment | 76 (60.8%) | 49 (39.2%) | 0.44 | 144 (%) | 518 (%) | 0.01 |
| Non-attached tick exposure or non-specific insect bite | 8 (47.1%) | 9 (52.9%) |  | 25 (%) | 76 (%) |  |
| No known exposure | 44 (55.7%) | 35 (44.3%) |  | 143 (28.5%) | 359 (71.5%) |  |
| Unknown | 260 (62.5%) | 156 (37.5%) |  | 486 (28.1%) | 1242 (71.9%) |  |
| **Exposure setting** |  |  |  |  |  |  |
| Home | 56 (50.9%) | 54 (49.1%) | 0.85 | 131 (25.5%) | 383 (74.5%) | <0.001 |
| Outdoors | 17 (56.7%) | 13 (43.3%) |  | 27 (21.8%) | 97 (78.2%) |  |
| Other | 79 (53.0%) | 70 (47.0%) |  | 151 (17.3%) | 720 (82.7%) |  |
| Unknown | 236 | 112 |  | 489 (33.0%) | 995 (67.0%) |  |
