# Supplementary material for: Spatial and clinical epidemiology of spotted fever rickettsioses and ehrlichiosis, North Carolina, 2010–2019
Source: PLoS Negl Trop Dis. 2025 Aug 13;19(8):e0013406. doi: 10.1371/journal.pntd.0013406 (PMC12364335; doi:10.1371/journal.pntd.0013406)
Supplement: S2 Table — (DOCX) [file pntd.0013406.s002.docx]

**Table 2.** **Spatial cluster categories and global Moran’s *I* values for ehrlichiosis and Spotted Fever Rickettsiosis (SFR) in North Carolina by ZIP code, 2010-2019**

| **Years** | **High** | **High outlier** | **Low** | **Low outlier** | **No cluster** | **Global Moran’s *I* (p)** |
| --- | --- | --- | --- | --- | --- | --- |
| **Ehrlichiosis** |  |  |  |  |  |  |
| 2010–2011 | 20 | 0 | 0 | 28 | 712 | 0.11 (<0.001) |
| 2012–2013 | 21 | 0 | 0 | 34 | 704 | 0.10 (<0.001) |
| 2014–2015 | 12 | 0 | 0 | 19 | 730 | 0.05 (<0.01) |
| 2016–2017 | 5 | 0 | 0 | 28 | 726 | 0.02 (0.17) |
| 2018–2019 | 17 | 0 | 0 | 29 | 714 | 0.07 (<0.001) |
| **Severe ehrlichiosis** |  |  |  |  |  |  |
| 2010–2019 | 7 | 0 | 0 | 4 | 304 | 0.12 (<0.01) |
| **SFR** |  |  |  |  |  |  |
| 2010–2011 | 18 | 0 | 0 | 13 | 730 | 0.06 (<0.001) |
| 2012–2013 | 47 | 0 | 0 | 25 | 687 | 0.29 (<0.001) |
| 2014–2015 | 46 | 0 | 0 | 16 | 697 | 0.21 (<0.001) |
| 2016–2017 | 22 | 0 | 0 | 5 | 732 | 0.21 (<0.001) |
| 2018–2019 | 42 | 0 | 0 | 16 | 701 | 0.17 (<0.001) |
| **Severe SFR** |  |  |  |  |  |  |
| 2010-2019 | 12 | 1 | 0 | 12 | 611 | 0.06 (<0.01) |
|  |  |  |  |  |  |  |
