# Supplementary material for: Spatial and clinical epidemiology of spotted fever rickettsioses and ehrlichiosis, North Carolina, 2010–2019
Source: PLoS Negl Trop Dis. 2025 Aug 13;19(8):e0013406. doi: 10.1371/journal.pntd.0013406 (PMC12364335; doi:10.1371/journal.pntd.0013406)
Supplement: S3 Table — (DOCX) [file pntd.0013406.s003.docx]

**Table 3. Sensitivity analysis of logistic regression models for predictors of severe ehrlichiosis and SFR in North Carolina, 2010-2019.** Primary models were adjusted models included multiple imputation for race, immunocompromised status and doxycycline treatment. Based on missingness analysis, the ehrlichiosis model included a delta-adjustment for immunocompromised status, and the Spotted Fever Rickettsiosis models included a delta adjustment for race.

| Independent variable | Adjusted OR* [95% CI] | p-value | Multiple imputation  model** OR [95% CI] | p-value |
| --- | --- | --- | --- | --- |
| Ehrlichiosis |  |  |  |  |
| Topographical region *(ref: Blue Ridge Mountain)* |  |  |  |  |
| Piedmont | 2.04 [0.85, 5.31] | 0.12 | 2.27 [0.94, 5.46] | 0.07 |
| Inner Coastal Plain | **2.83 [1.05, 8.20]** | **0.05** | **3.15 [1.17, 8.51]** | **0.02** |
| Tidewater | 2.25 [0.80, 6.73] | 0.07 | 2.34 [0.85, 6.43] | 0.10 |
| Likely exposure setting *(ref: unknown)* |  |  |  |  |
| Home | 0.58 [0.32, 1.04] | 0.07 | 0.64 [0.37, 1.09] | 0.10 |
| Outdoors | 0.74 [0.29, 1.88] | 0.5 | 0.82 [0.51, 1.33] | 0.42 |
| Other | 0.74 [0.44, 1.24] | 0.8 | 0.96 [0.40, 2.33] | 0.93 |
| Known tick exposure *(ref: no known tick exposure)* |  |  |  |  |
| Tick attachment | 1.29 [0.63, 2.67] | 0.5 | 1.36 [0.68, 2.71] | 0.39 |
| Non-attached tick exposure or non-specific insect bite | 0.40 [0.09, 1.55] | 0.2 | 0.70 [0.21, 2.36] | 0.56 |
| Unknown | 1.07 [0.58, 1.97] | 0.8 | 1.15 [0.64, 2.05] | 0.64 |
| Any antibiotic treatment *(ref: no antibiotic treatment)* | 2.09 [0.90, 5.17] | 0.09 | 2.21 [0.94, 5.21] | 0.07 |
| Doxycycline treatment *(ref: no doxycycline treatment)* | 0.83 [0.43, 1.58] | 0.6 | 0.840 [0.44, 1.60] | 0.59 |
| Doxycycline treatment delay |  |  |  |  |
| *From specimen date (ref: same day)* |  |  |  |  |
| 1–7 days post specimen date | 0.92 [0.43, 1.94] | 0.8 | 1.41 [0.70, 2.83] | 0.33 |
| >7 days post specimen date | **0.27 [0.08, 0.76]** | **0.02** | **0.35 [0.13, 0.96]** | **0.04** |
| *From illness identification date (ref: 0-3 days)* |  |  |  |  |
| 4–7 days post symptom onset | 2.49 [1.00, 6.46] | 0.06 | 1.88 [0.97, 3.65] | 0.06 |
| 8–14 days post symptom onset | 2.39 [0.85, 7.05] | 0.11 | 1.08 [0.50, 2.31] | 0.85 |
| >14 days post symptom onset | 1.10 [0.40, 3.02] | 0.9 | 0.80 [0.36, 1.74] | 0.57 |
| Antibiotic treatment course *(ref: doxycycline only)* |  |  |  |  |
| No doxycycline treatment | 1.57 [0.80, 3.11] | 0.2 | 1.53 [0.78, 2.99] | 0.21 |
| Multiple antibiotics with delayed doxycycline | 1.57 [0.38, 7.00] | 0.5 | 1.69 [0.42, 6.81] | 0.460 |
| Multiple antibiotics with immediate doxycycline | **18.6 [4.95, 122]** | **<0.001** | 18.65 [4.08, 85.33] | <0.001 |
| Spotted Fever *Rickettsia* |  |  |  |  |
| Topographical region *(ref: Blue Ridge Mountain)* |  |  |  |  |
| Piedmont | 0.95 [0.64, 1.43] | 0.8 | 1.06 [0.75, 1.49] | 0.73 |
| Inner Coastal Plain | 1.04 [0.65, 1.67] | 0.9 | 1.14 [0.77, 1.69] | 0.52 |
| Tidewater | 1.10 [0.68, 1.80] | 0.7 | 1.25 [0.82, 1.89] | 0.29 |
| Likely exposure setting *(ref: unknown)* |  |  |  |  |
| Home | **0.69 [0.50, 0.94]** | **0.02** | **0.62 [0.47, 0.82]** | **<0.001** |
| Outdoors | **0.55 [0.42, 0.73]** | **<0.001** | **0.45 0.35, 0.57]** | **<0.001** |
| Other | 0.63 [0.35, 1.09] | 0.11 | **0.51 [0.30, 0.86]** | **0.01** |
| Known tick exposure *(ref: no known tick exposure)* |  |  |  |  |
| Tick attachment | **0.61 [0.43, 0.86]** | **<0.01** | **0.67 [0.48, 0.93]** | **0.02** |
| Non-attached tick exposure or non-specific insect bite | 0.55 [0.27, 1.06] | 0.09 | 0.71 [0.39, 1.29] | 0.25 |
| Unknown | **0.71 [0.53, 0.96]** | **0.02** | 0.94 [0.72, 1.23] | 0.67 |
| Any antibiotic treatment *(ref: no treatment)* | 1.13 [0.68, 1.96] | 0.5 | 1.17 [0.69, 1.97] | 0.56 |
| Doxycycline treatment *(ref: no doxycycline)* | **0.62 [0.43, 0.89]** | **<0.01** | **0.66 [0.46, 0.95]** | **0.02** |
| Doxycycline treatment delay |  |  |  |  |
| *From specimen date (ref: same day)* |  |  |  |  |
| 1–7 days post specimen date | **1.54 [1.10, 2.14]** | **0.01** | **1.54 [1.14, 2.08]** | **0.01** |
| >7 days post specimen date | 0.76 [0.37, 1.44] | 0.3 | 0.96 [0.54, 1.71] | 0.09 |
| *From illness identification date (ref: 0-3 days)* |  |  |  |  |
| 4–7 days post symptom onset | 1.07 [0.70, 1.61] | 0.8 | 1.06 [0.67, 1.70] | 0.80 |
| 8–14 days post symptom onset | 1.51 [0.93, 2.43] | 0.09 | 1.36 [0.92, 2.01] | 0.13 |
| >14 days post symptom onset | 1.09 [0.61, 1.89] | 0.8 | 1.15 [0.84, 1.57] | 0.38 |
| Antibiotic treatment course *(ref: doxycycline only)* |  |  |  |  |
| No doxycycline treatment | **1.94 [1.33, 2.79]** | **<0.001** | **1.78 [1.24, 2.56]** | **<0.001** |
| Multiple antibiotics with delayed doxycycline | 5.06 [2.23, 11.7] | **<0.001** | **5.15 [2.38, 11.15]** | **<0.001** |
| Multiple antibiotics with immediate doxycycline | **5.49 [2.98, 10.3]** | **<0.001** | **5.13 [2.87, 9.18]** | **<0.001** |

*All models in this table included adjustment for age, race immunocompromised status. Topographical region, likely exposure setting and known tick exposure models also included adjustment for any doxycycline treatment.

**Multiple imputation performed for race, immunocompromised status and doxycycline treatment. Ehrlichia models used delta-adjustment for immunocompromised, and SFR models used delta-adjustment for race. Delta adjustment were 0.4 on the log odds scale, corresponding to an odds ratio of approximately 1.5, assuming a moderate level of unmeasured confounding.
