# Supplementary material for: Spatial and clinical epidemiology of spotted fever rickettsioses and ehrlichiosis, North Carolina, 2010–2019
Source: PLoS Negl Trop Dis. 2025 Aug 13;19(8):e0013406. doi: 10.1371/journal.pntd.0013406 (PMC12364335; doi:10.1371/journal.pntd.0013406)
Supplement: S1 Fig — (DOCX) [file pntd.0013406.s004.docx]

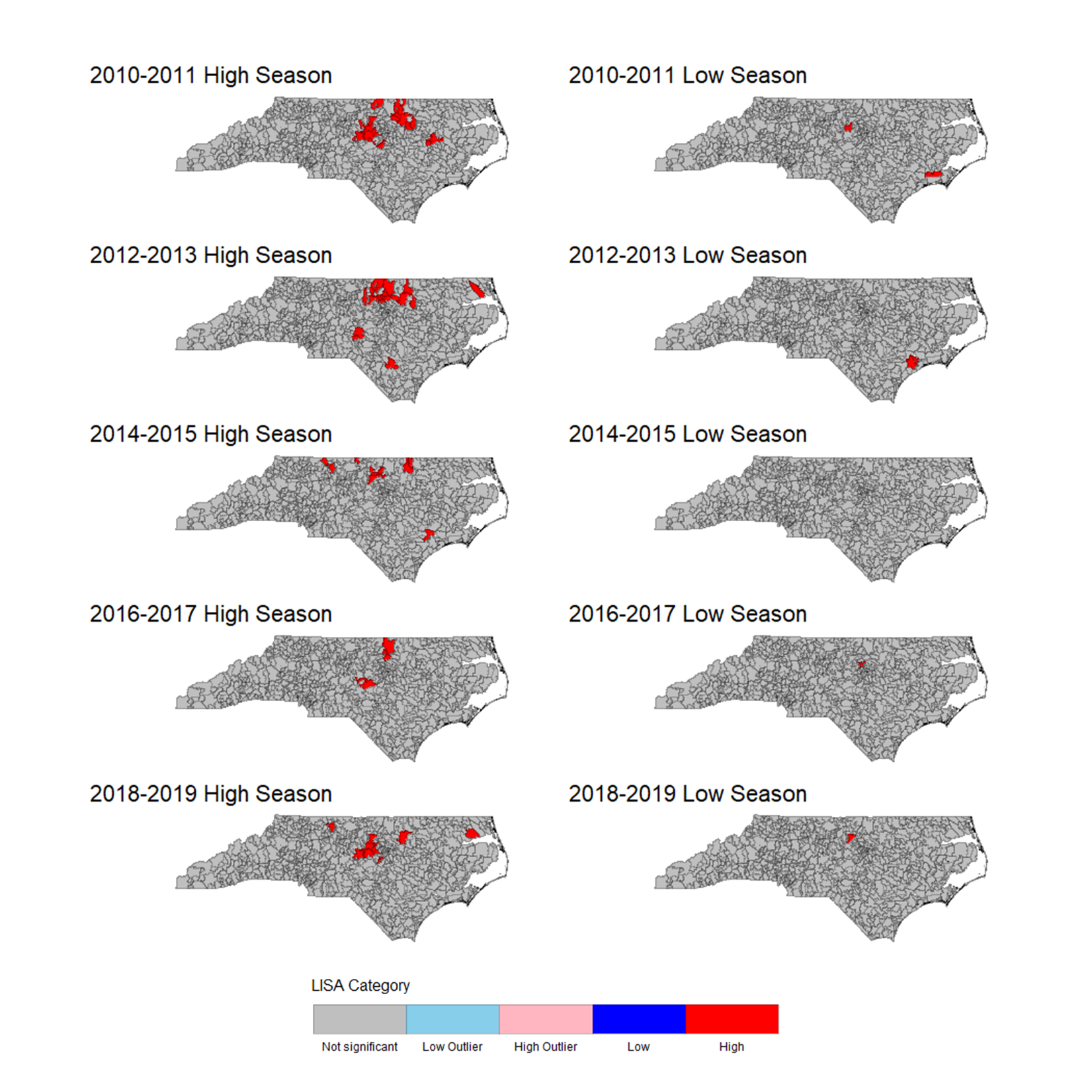
**Fig 1.** **Biannual high season (April through October) and low season (November through March) clusters of ehrlichiosis in North Carolina by ZIP code, 2010-2019.** Maps generated using a TIGER/Line shapefile available from the US Census Bureau, https://www.census.gov/geographies/mapping-files/time-series/geo/tiger-line-file.html
